# Detection and analysis of alternative splicing in *Yarrowia lipolytica *reveal structural constraints facilitating nonsense-mediated decay of intron-retaining transcripts

**DOI:** 10.1186/gb-2010-11-6-r65

**Published:** 2010-06-23

**Authors:** Meryem Mekouar, Isabelle Blanc-Lenfle, Christophe Ozanne, Corinne Da Silva, Corinne Cruaud, Patrick Wincker, Claude Gaillardin, Cécile Neuvéglise

**Affiliations:** 1 INRA UMR1319 Micalis - AgroParisTech, Biologie intégrative du métabolisme lipidique microbien, Bât. CBAI, 78850 Thiverval-Grignon, France; 2 Genoscope (CEA) - Centre National de Séquençage, 2 rue Gaston Crémieux, 91057 Evry cedex, France

## Abstract

**Background:**

Hemiascomycetous yeasts have intron-poor genomes with very few cases of alternative splicing. Most of the reported examples result from intron retention in *Saccharomyces cerevisiae *and some have been shown to be functionally significant. Here we used transcriptome-wide approaches to evaluate the mechanisms underlying the generation of alternative transcripts in *Yarrowia lipolytica*, a yeast highly divergent from *S. cerevisiae*.

**Results:**

Experimental investigation of *Y. lipolytica *gene models identified several cases of alternative splicing, mostly generated by intron retention, principally affecting the first intron of the gene. The retention of introns almost invariably creates a premature termination codon, as a direct consequence of the structure of intron boundaries. An analysis of *Y. lipolytica *introns revealed that introns of multiples of three nucleotides in length, particularly those without stop codons, were underrepresented. In other organisms, premature termination codon-containing transcripts are targeted for degradation by the nonsense-mediated mRNA decay (NMD) machinery. In *Y. lipolytica*, homologs of *S. cerevisiae UPF1 *and *UPF2 *genes were identified, but not *UPF3*. The inactivation of *Y. lipolytica UPF1 *and *UPF2 *resulted in the accumulation of unspliced transcripts of a test set of genes.

**Conclusions:**

*Y. lipolytica *is the hemiascomycete with the most intron-rich genome sequenced to date, and it has several unusual genes with large introns or alternative transcription start sites, or introns in the 5' UTR. Our results suggest *Y. lipolytica *intron structure is subject to significant constraints, leading to the under-representation of stop-free introns. Consequently, intron-containing transcripts are degraded by a functional NMD pathway.

## Background

From a genomic point of view *Yarrowia lipolytica *is rather atypical among hemiascomycetous yeasts sequenced to date [1]. Its genome is surprisingly large, consisting of six chromosomes, a total of about 20.5 Mb in size, more than one and a half times the size of the *Saccharomyces cerevisiae *genome and twice that of *Kluyveromyces lactis*. However, with an overall density of only one gene per 3 kb and 6,449 predicted protein-coding genes, the gene content of *Y. lipolytica *is similar to that of other hemiascomycetes. The complete genome has a mean G + C content of 49%, which is significantly higher than that in other yeast genomes [1,2], with the exception of *Eremothecium *(*Ashbyia*) *gossypii*, which has a G + C content of 52% [3]. The genome of *Y. lipolytica *is also unusual in several other ways: atypical structure of chromosomal origins of replication and centromeric DNA [4], large number of tRNA genes [1,5], 5S rRNA genes dispersed throughout the genome [1,6] and unique fusions between tRNA genes and 5S rRNA genes [7]. Unlike most hemiascomycetes, in which ribosomal DNA loci are clustered into a single locus on one chromosomal arm, *Y. lipolytica *rDNA units, containing the 18S, 5.8S and 26S rRNA genes, are found in six subtelomeric clusters [1,8], a distribution also observed in *Pichia pastoris *[9]. *Y. lipolytica *is also unusual in having a highly diverse transposable element content [10-13]. *Y. lipolytica *genes also display an organization different from that of other hemiascomycetes, as some genes are interrupted by several spliceosomal introns, with up to five introns per gene [1,14]. The total number of introns, first estimated at 742 in the 2004 annotation, has now reached 1,119 with the data presented in this study, and this number of introns is larger than that in any other hemiascomycetous genome sequenced to date (287 introns in *S. cerevisiae *[15]; 415 introns in *Candida albicans *[16]; 633 intron-containing genes in *P. pastoris *[9]). Thus, about 15% of the genes contain introns and the intron density is about 0.17.

Intron density varies considerably between eukaryotes [17], from a few introns per genome in *Giardia *[18], to more than eight per gene in humans [19]. *Y. lipolytica *is thus considered to be an intron-poor species [20], but alternative splicing (AS) was fortuitously observed for the intron of the first gene of the Mutyl DNA transposon, for which a combination of alternative 5'-splice sites (5'ss) and 3'-splice sites (3'ss) is used [13]. AS generally results from the combination of splice sites present in the pre-mRNA, and may occur through four basic modes: use of an alternative 5'ss, use of an alternative 3'ss, cassette-exon skipping and intron retention. AS is currently thought to occur in more than 60% of human genes [21-23], increasing the complexity of the transcriptome and leading to genetic or malignant diseases in some cases [24,25]. By contrast, very few examples of AS resulting in the production of multiple proteins have been reported in yeasts, such as *Schizosaccharomyces pombe *[26] and *S. cerevisiae *[27,28]. In a few additional cases, alternative transcripts have been predicted in *S. cerevisiae *[29-31] and *C. albicans *[16] although without supporting evidence for multiple functional proteins. Many other cases of alternative transcripts in yeasts, mostly identified by global transcriptomic approaches [32-34], involve intron retention and result in nonsense-containing mRNAs. These cases may result from inefficient splicing or missplicing [35] due to suboptimal splicing signals [36]. These alternative transcripts were thought to be largely non-functional. However, in some cases, intron retention seems to be regulated by growth conditions, such as amino-acid starvation [37], or by a specific physiological state of the cells, such as meiosis [15,38,39]. Other examples of regulated splicing, in which the protein inhibits the splicing of its own pre-mRNA, include RPL30 [40] and YRA1 [27,41,42].

Thus, the AS of mRNA generates two types of transcript: mRNAs to be translated into functional proteins (thereby increasing the complexity/diversity of the proteome) or nonsense-containing mRNAs that may generate truncated proteins potentially deleterious to the cell if translated. Nonsense-mediated mRNA decay (NMD) is a eukaryotic quality control mechanism that detects mRNAs with a premature termination codon (PTC), targeting them for degradation and thus preventing their translation (for review, see [43-45]). This RNA surveillance pathway is well documented in yeast, mammals, fruit flies, nematodes and plants [46,47]. Different mechanisms of PTC recognition have been identified in different species, involving the exon-exon junction complex in mammals, and the distance between the PTC and the poly(A)-binding protein, also called the 'faux 3' UTR', in yeast and fruit fly [48]. However, a unified model has also been proposed in recent studies [49].

When introns are retained, a PTC may be generated by the intron sequence itself or by the downstream exon sequence if the intron does not consist of a multiple of three nucleotides and thus generates a frameshift. This observation led Jaillon *et al. *[50] to suggest that introns are structured so as to favor their detection by the NMD pathway in cases of intron retention. These authors showed that, in different species from very different phyla, intron size was subjected to strong constraints leading to the counterselection of stop-less introns of size 3n (that is, consisting of a multiple of three nucleotides).

The mechanisms regulating AS and NMD are not fully understood. Yeasts are tractable unicellular models that could supply molecular information about such mechanisms. As *Y. lipolytica *has more introns than *S. cerevisiae*, it is likely to display more AS and thus to be useful for investigation of the associated molecular mechanisms. We therefore investigated, in this organism, the population of transcripts from intron-containing genes, and their likelihood of degradation by the NMD pathway, through a combination of several different experimental approaches.

## Results

### cDNA sequencing shows *Y. lipolytica *to have four times as many introns as *S. cerevisiae*

We began our investigation of *Y. lipolytica *splicing by using cDNA sequencing to revisit the *in silico *predictions of intron-containing genes in this yeast. Three cDNA libraries were constructed from mRNAs obtained from cells grown under different conditions: exponential and stationary phases on YPD medium ('expo', 9,409 reads; and 'stat', 9,620 reads) and exponential phase on oleic acid medium ('oleic', 9,405 reads).

We found that 1,659 of the 28,434 cDNA sequences (5.8%) did not match the predicted coding sequence (CDS), with 455 of these sequences not matching the *Y. lipolytica *chromosome sequence but possibly corresponding to CDS in non-assembled contigs. Some of the remaining 1,204 non-matching sequences displayed a significant match with 21 of the 137 predicted pseudogenes in the sense (64 cDNA sequences) or anti-sense (22 cDNA sequences) orientation. The others corresponded to intergenic regions with no predicted genetic elements.

Another set of 1,053 cDNA sequences (3.7%) matched, in an anti-sense orientation, with 167 *Y. lipolytica *CDSs, one of which (YALI0A21351g) was highly represented, with 579 cDNA clones. YALI0A21351g has been predicted to encode a small gene product (89 amino acids) with no homolog in databases, and may therefore be a false open reading frame. The cDNA clones derived from the antisense transcripts may thus correspond to a non-coding RNA, the structure and function of which remain to be determined.

We found that 25,722 clones matched a CDS in the expected orientation: 8,936, 8,614 and 8,172 clones in the expo, stat and oleic libraries, respectively. About 59% of the predicted genes (3,818 of 6,449) were expressed and found in at least one library and about 70% of these expressed genes (2,647 genes) were represented by at least two different clones. Clone numbers per gene and per library are given in Additional file 1. A few genes (13 genes) were represented by more than 100 clones, but mostly by less than 200, in the different libraries. The major exceptions were YALI0D06237g and YALI0E15510g in the stat library, which had 713 clones (8.7% of the stat clones) and 679 clones (8.3% of the stat clones), respectively. YALI0D06237g encodes a putative sphingolipid delta 4 desaturase and YALI0E15510g a putative homeobox transcriptional repressor. Comparison between the cDNA sequences of the different libraries showed that only 20% of the sequenced cDNAs were expressed in all three growth conditions (Figure S1 in Additional file 2). About 12% of the sequenced cDNAs were specific to the oleic or stat libraries, but almost twice as many (22.6%) were specific to the expo library. However, these figures are only approximations, as cDNA library sequencing is certainly not the most sensitive way to quantify gene expression. Some overlap in expression patterns between the different conditions may therefore have been missed due to low levels of expression or cloning biases.

Based on the cDNA data, the information in the genome database concerning start codon coordinates, the presence or absence of introns and intron coordinates, when already predicted, was modified. New genes were also detected, including three genes specifically induced on oleic acid medium (*SOA1*, *SOA2 *and *SOA3 *genes [51]). In total, 6,449 protein-coding genes are now predicted for *Y. lipolytica *strain E150 (Table 1). Gene model modifications are reported in the Génolevures database [52].

**Table 1:** Distribution of introns and intron-containing genes in the E150 genome

			Intron-containing genes (I-genes) with:						
Chromosome	Genes	Pseudo-genes	1 intron	2 introns	3 introns	4 introns	5 introns	Total I-genes	Total introns
YALI0A	686	32	66 (6)	8	0	0	0	74 (6)	82 (6)
YALI0B	949	14	138 (6)	17	2	1	0	158 (6)	182 (6)
YALI0C	932	30	133 (6)	11 (1)	3	0	1	148 (7)	169 (8)
YALI0D	1,101	29	131 (6)	20	1	1	0	153 (6)	178 (6)
YALI0E	1,464	12	177 (4)	18	1	0	0	196 (4)	216 (4)
YALI0F	1,317	20	197 (2)	19 (2)	4	1	1	222 (4)	256 (6)
Genome	6,449	137	842 (29)	93 (3)	11	3	2	951 (33)	1,083 (36)


Introns were detected in 5' UTRs. The number of 5' UTR introns or of genes containing 5' UTR introns is indicated in parentheses.

The number of predicted introns in the sequenced E150 genome increased from 742 [1] to 1,083, and the number of intron-containing genes increased to 951. Most of these genes carry only one intron, but 109 multi-intronic genes with up to five introns were detected, most (93 of 109) carrying two introns (Table 1). The internal exons of the multi-intronic genes were mostly short, the shortest being only four nucleotides long, in YALI0E34170g, as validated by two cDNAs. Introns in 5' UTRs were not systematically predicted during *in silico *annotation by the Génolevures Consortium. Our data revealed the presence of at least 36 introns in these 5' non-coding regions of mRNAs, a number similar to that reported for *S. cerevisiae *[31]. Thus, with 1,119 introns, *Y. lipolytica *is the hemiascomycete with the largest number of spliceosomal introns in its genome, with about four times as many introns as *S. cerevisiae*.

### *Y. lipolytica *introns have several unique features

**Figure 1:**
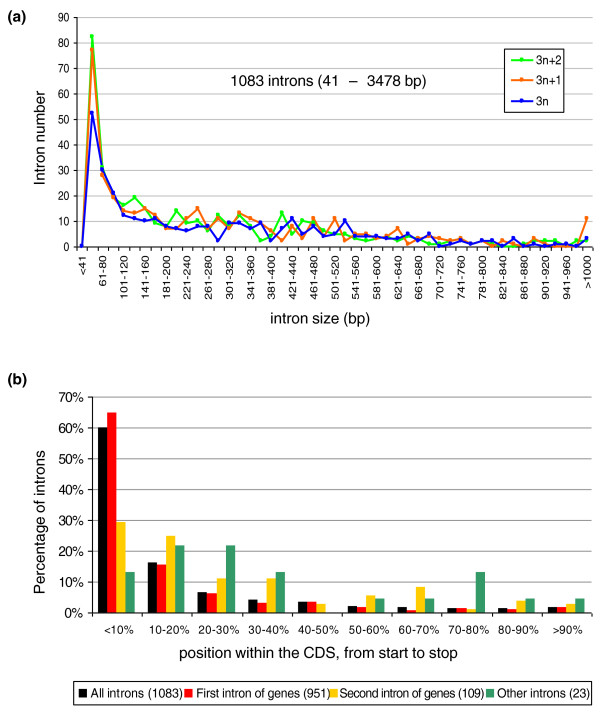
**Characteristics of *Y. lipolytica *introns**. **(a) **Size distribution of the 1,083 introns from strain E150 located within the coding regions of genes. Introns are separated into three size classes: multiples of 3 nucleotides (blue line), multiples of 3 plus 1 nucleotides (orange line), and multiples of 3 plus 2 nucleotides (green line). For each class, the number of introns is reported as a function of size, with a window of 20 nucleotides from 41 nucleotides to more than 1,000 nucleotides. **(b) **Position of introns within the CDS. Introns are separated according to their order in the gene model, from start to stop: first introns of genes (red boxes), second introns of genes (orange boxes) and other introns (green boxes). Data for all introns considered together are shown in black. The proportion of introns in each group is plotted as a function of their relative position within the CDS, with a window of 10% of the CDS length.

Intron size in *Y. lipolytica *varies from 41 to 3,478 bp (16 introns were larger than 1 kb), with a mean length of 280 bp and a median length of 204 bp. This is a broader range of sizes than observed in other yeasts, in which the maximum intron size is usually around 1 kb (1,002 bp for *S. cerevisiae*). However, the intron size distribution is biased toward short introns (33% of introns are less than 100 bp long), with a dominant peak distribution between 41 and 60 nucleotides (Figure 1a). This bias has previously been observed in other fungi, such as *S. pombe *and *Neurospora crassa *[53]. As previously reported in other hemiascomycetes [54] and in some intron-poor eukaryotic genomes [55,56], the position of introns in the coding sequence was also biased. About 60% of all introns were inserted in the first 10% of the CDS (Figure 1b) and this figure rose to 65% if only the first intron was considered. For example, 47 genes had a first coding exon of only one base, the adenine of the methionine initiation codon. We also detected 36 introns in the 5' UTRs of 33 genes, all but four of which had no introns in their coding sequences. Most of these 5' UTR introns were validated by cDNA sequencing (Additional file 3). They were generally larger than the introns in coding regions (Figure S2a in Additional file 2), with only five 5' UTR introns less than 100 bp in length (approximately 14% of the 5' UTR introns). We validated this greater intron length by simulations: among 100 randomly generated sets of 36 introns chosen among the 1,083 introns, none presented a mean length equal or superior to that of the 5' UTR introns (the maximum mean length was 381 bp; Additional file 4). Size differences between the introns found in coding sequences and those in 5' UTRs have already been reported for various eukaryotes, including humans, mice, *Drosophila melanogaster *and *Arabidopsis thaliana *[57].

Several unique features were identified when the intron structure of *Y. lipolytica *was compared with that of other hemiascomycetous yeasts. First, the branch point (BP) and the 3'ss were found to form a combined sequence, with a mean interval of one nucleotide between the motifs (Figure S2a,b in Additional file 2). This finding was previously reported for a small subset of introns of strain W29 [14] and for a larger subset of introns of *Y. lipolytica *sequenced strain [58,59]. This juxtaposition may result from an evolutionary event that simplified the mechanism of spliceosomal assembly, combining the steps of BP and 3'ss recognition [58], as hypothesized for two other deep-branch eukaryotes, *Trichomonas vaginalis *and *Giardia lamblia *[18]. Second, the consensus sequences at intron boundaries were also found to be unusual for yeasts. This was particularly true for the 5'ss, which had the sequence GTGAGT, rather than the GTATGT sequence found in most other hemiascomycetes [14,58,60,61]. This 5'ss consensus, which is known to be essential for intron recognition by base-pairing to U1 snRNAs, is indeed perfectly complementary to both *Y. lipolytica *U1 RNAs (YALI0B14567r and YALI0B20936r; Figure S3 in Additional file 2). Third, the internal BP is less well conserved than in other hemiascomycetes sequenced to date, with only five highly conserved residues (CTAAC in more than 92% of the introns) and an upstream A less conserved (Actaac in more than 71%; Figure S2A in Additional file 2), rather than the seven (TACTAAC) reported for *S. cerevisiae *[61].

All intron patterns and sequences can be downloaded from the Génosplicing website [62].

### Structural biases in *Y. lipolytica *introns

We investigated the distribution of introns as a function of the translation frame of upstream exons (an intron is considered to be in phase 0 if located between two codons and in phase 1 or 2 if it splits a codon after the first or second nucleotide, respectively), intron size and the number of in-frame stop codons. This analysis highlighted several constraints exerted on the introns interrupting CDS.

**Figure 2:**
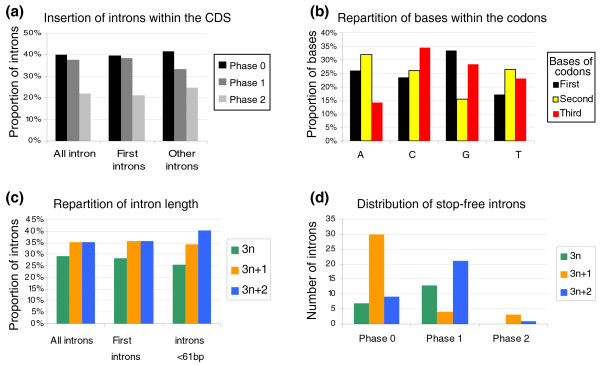
**Distribution of introns as a function of their length and insertion frame**. **(a) **Introns are represented according to the three possible frames of the CDS. Phase 0 indicates that the intron is located between two codons, phase 1 indicates that it is located after the first nucleotide of a codon and phase 2 indicates that it is located after the second nucleotide of a codon. 'All introns' corresponds to the 1,083 introns, 'first introns' to the first intron of the 951 intron-containing genes and 'other introns' to the 131 second, third, fourth and fifth introns of genes. Differences between insertion phases were statistically significant for all introns (c^2 ^= 64.68, *P *= 8.98e-15) or for the first introns (c^2 ^= 60.68, *P *= 6.63e-14) but not for introns other than the first intron (c^2 ^= 5.50, *P *= 0.063), probably due to their limited number. **(b) **The proportions of each of the four bases are represented for each base of the codons of the 6,449 protein-coding genes. Differences in nucleotide distribution were statistically significant for each position within the codon (c^2 ^test, *P *<< e-100). Stop codons were not considered. **(c) **Introns shown according to length categories, corresponding to a multiple of 3 (3n) or a multiple of 3 plus 1 nucleotides (3n + 1) or plus 2 nucleotides (3n + 2). There were 204 introns ≤60 nucleotides in length. The underrepresentation of 3n introns was statistically significant for all introns (c^2 ^= 7.35, *P *= 0.025), first introns (c^2 ^= 10.90, *P *= 0.004) and for introns no longer than 60 nucleotides (c^2 ^= 6.70, *P *= 0.034). **(d) **Stop-free introns are represented according to their insertion frame and length category.

First, as previously reported for various eukaryotes [63,64] most introns were inserted in phase 0 (40.2% of all introns) or phase 1 (38%), with a highly significant underrepresentation of intron insertions in phase 2 (21.8%; c^2 ^= 64.68, *P *= 8.98e^-15^; Figure 2a). The nucleotide environment of the 5'ss has a strong impact on the efficiency of base-pairing to the U1 snRNA, and the nucleotide upstream of the 5'ss is particularly important [65,66]. In *Y. lipolytica*, this nucleotide is generally a guanosine (48.5%; Figure S2a in Additional file 2), as also reported for *S. cerevisiae *[67]. We looked for a correlation between intron phase and the presence of G residues upstream of introns by determining codon usage for the 6,449 genes of *Y. lipolytica*. We found that G residues were less frequent in position two within the codon than in positions one and three (Figure 2b), potentially accounting for the observed bias in favor of phase 0 and phase 1 introns.

Second, introns of size 3n were underrepresented (29.4% of all introns versus 35.5% and 35.1% for 3n + 1 and 3n + 2, respectively; Figure 2c). This observation is consistent with the finding that stop-less 3n introns are counterselected in *Paramecium tetraurelia *[50]. In *Y. lipolytica*, the underrepresentation of 3n introns seemed more marked if we considered only the first intron (28.3% versus 35.85% for each 3n + 1 and 3n + 2 intron), or if we considered only short introns of 41 to 60 nucleotides (25.5% versus 34.3% and 40.2% for 3n + 1 and 3n + 2 introns, respectively; Figure 1a). No statistically significant difference was found in the distribution of introns present in the 5' UTR: 11, 13 and 12 introns of size 3n, 3n + 1 and 3n + 2, respectively (Additional file 3).

Third, the proportion of introns containing in-frame stop codons was very high for 3n (93.7%), 3n + 1 (90.4%) and 3n + 2 introns (91.8%). The probability of an intron not containing a PTC (null expectation) in a non-constrained codon string is smaller than 0.05% for any string longer than 62 codons (Figure S4 in Additional file 2). We thus compared the distribution of PTCs in introns shorter than 186 nucleotides with the expected probability. The proportion of stop-containing introns was higher than would be expected by chance alone (Figure S4 in Additional file 2). Thus, stop-free introns are scarce (88 stop-free introns). Their distribution as a function of length and insertion frame was highly heterogeneous, with an overrepresentation of stop-free 3n + 1 introns inserted in phase 0 and of 3n + 2 introns in phase 1 (Figure 2d).

**Figure 3:**
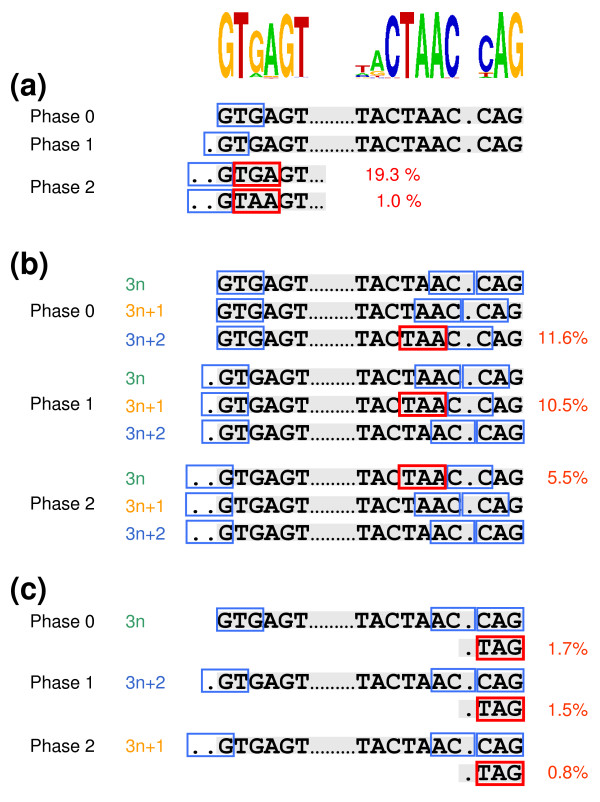
**Presence of premature termination codons in spliceosomal introns, as a function of intron size (3n, 3n + 1, 3n + 2) and insertion frame (frame 0, 1 and 2) within the coding sequence**. **(a) **A PTC is generated for all retained introns inserted in frame 2 and containing GTGAGT or GTAAGT as the 5'ss sequence, whatever their length; 209 introns are concerned, that is, 19.3% of all intron-containing genes. **(b) **PTCs (TAA) are also detected in the BP of 3n + 2 introns in frame 0, 3n + 1 introns in frame 1 or 3n introns in frame 2 if the S2 distance is indeed 1 bp. **(c) **The main 3'ss is CAG, but, in about 10.5% of the introns, TAG is also used. This sequence generates a PTC for 3n introns inserted in frame 0, 3n + 2 introns in frame 1 and 3n + 1 introns in frame 2. Overall, conserved intron motifs are present in about 50% of the PTC-containing introns.

We hypothesized that the unusual intron boundaries in *Y. lipolytica *might account for the high frequency of PTCs in short introns. The 5'ss motif GTGAGT generates an in-frame stop codon in introns inserted in phase 2, whatever their size, and this situation applied to 209 introns (19.3% of the 1,083 introns; Figure 3a). Similarly, GTAAGT, the second most frequent motif, was responsible for 1% (11 introns) of stop-containing introns in phase 2. The conserved part of the BP motif, CTAAC, also generated stop codons. Assuming that the distance between the BP and 3'ss motifs (S2 distance) is a mean of one base (Figure S2 in Additional file 2), three categories of introns (phase 0 size 3n + 2, phase 1 size 3n + 1 and phase 2 size n) are most likely to contain in-frame stop codons in BP. Indeed, 125, 114 and 60 introns, respectively, fell into these categories (27.6% of all introns; Figure 3b). The involvement of the BP motif is clearly underestimated, as the S2 distance may be different from one base (possibly shorter or longer than one base), making it possible for introns inserted in other phases to contribute to the presence of an in-frame stop codon in the BP motif. Finally, the 3'ss TAG is also responsible for the generation of 4% of stop codons (Figure 3c). These consensus sequences together account for at least 50% of stop codons. Thus, the constraints exerted on donor, acceptor and BP motifs are not only necessary for splicing (intron definition mechanism) but, together with constraints on intron size and phasing within the codons, also contribute to intron modeling.

### *Y. lipolytica *uses all modes of alternative splicing

AS events were sought by two different experimental approaches. First, transcripts of genes with multiple introns or with large introns (>900 bp) were investigated by RT-PCR. Subsequently, sequences obtained from cDNA libraries were screened for splicing variants.

#### Multi-intronic genes

RT-PCR was carried out on 93 genes of *Y. lipolytica *for which *in silico *predictions for more than one intron had been made at the beginning of this study (Additional file 5). For 68 of these genes, the predicted spliced transcript was confirmed and a single mRNA was detected. Two other gene models (YALI0F03817g and YALI0F31427g) were poorly predicted and, in both cases, the second intron was not spliced in any of the three RNA preparations. It was thus considered to be part of an exon, resulting in a monointronic gene model. In nine RT-PCRs, no result was obtained, due to an absence of PCR product or non-specific amplification. For two other predicted gene models (YALI0C07150g and YALI0D04554g), only partial data were obtained and we were able to confirm only the splicing of intron 2.

**Figure 4:**
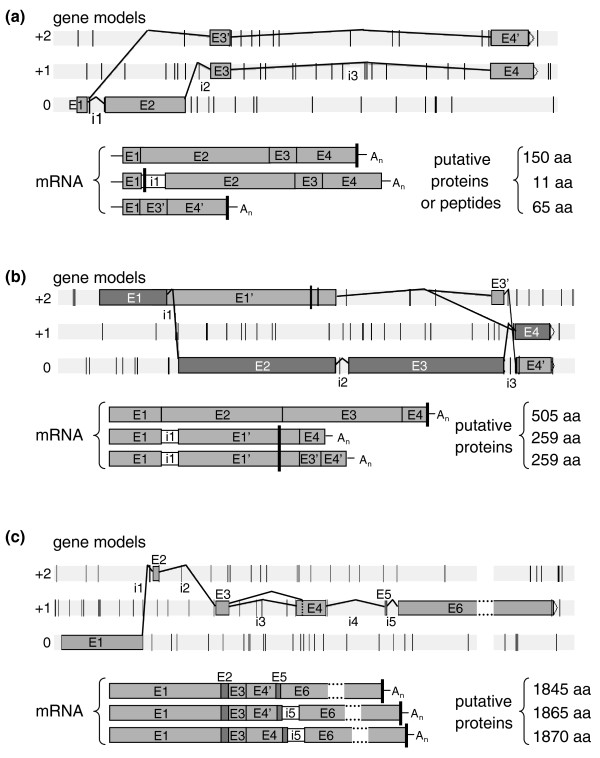
**Schematic representation of alternative transcripts from multi-intronic genes**. Gene models include exons, represented by gray rectangles and introns, symbolized by thin black articulated lines. Vertical bars on each of the three phases (0, +1 and +2) represent an in-frame stop codon. The resulting mRNA variants are depicted as a concatenation of exons and the thick black vertical line represents the first in-frame codon of the transcript. The size of the putative proteins derived from each splicing variant is indicated on the right. All three genes generate at least three different splicing variants. **(a) **YALI0C23496g mRNAs are subject to intron retention (intron 1) or exon skipping (exon 2). The retention of intron 1 generates a PTC and a putative peptide of 11 amino acids. Exon 2 skipping generates a frameshift in exon 3 and in exon 4, which is slightly shortened (exon 4'), and generates a putative protein of 65 amino acids. **(b) **YALI0F26873g splicing variants display retained intron 1, alternative 3'ss (intron 2) usage or the skipping of exon 3. Both variants with a retained intron 1 generate a PTC in exon 2 and a putative truncated protein of 259 amino acids. **(c) **In YALI0F32043g mRNAs, the retention of intron 5 and the use of an alternative 3'ss do not generate a PTC or a frame shift in that intron 5 is a multiple of three (60 nucleotides) nucleotides long and the difference between E4 and E4' is also a multiple of three (15 nucleotides). Both variants generate a putative protein of about the same size as that generated by the fully spliced transcript. Considering the large size of exon 6, it is shown truncated with horizontal dashed lines.

The last 12 RT-PCRs revealed the presence of multiple transcripts, corresponding to different splicing variants. For nine of these genes, we observed both transcripts with retained introns, and transcripts efficiently spliced. For seven of these transcripts, only the first intron of the gene was retained, whereas, in one case (YALI0F16753g), either intron 1 or 2 was retained and, in the last case (YALI0C15323g), only the second intron was retained. The last three cases involved both intron retention and exon skipping events. For YALI0C23496g, we observed either intron 1 retention, introducing a PTC after 11 codons, or exon 2 skipping, changing the phase of exons 3 and 4 and generating different putative proteins (Figure 4a). For YALI0F26873g, two mRNA variants were detected in addition to the predicted fully spliced transcript responsible for generating the putative 505 amino acid protein (Figure 4b). In both alternative transcripts, exon 3 was skipped either totally (splicing between 5'ss of intron 2 and 3'ss of intron 3) or partially (alternative 3'ss of intron 2, leaving 45 nucleotides of exon 3). Both variants retained the stop-free intron 1, which changed the predicted phase and generated a PTC within exon 2, thereby resulting in a truncated 259 amino acid protein. This gene belongs to the large septin family, which has seven members in *Y. lipolytica*, as in most hemiascomycetous yeasts. Surprisingly, all but one of the genes in this family contain at least one intron, the splicing of which was validated by cDNA clones. YALI0F26873g is the only gene of this family with three introns and the only member of the family with alternative transcripts. Mitrovich *et al. *[16] observed that three of the seven septins of *C. albicans *contained introns and suggested that AS might play an important role in their regulation, consistent with our findings.

#### Genes bearing long introns (>900 bp)

Long introns are rare in *S. cerevisiae*, with all but five of the introns in this species being less than 700 nucleotides long and the largest intron being 1,002 bp long. In *Y. lipolytica*, gene model predictions indicate that there are 61 introns of more than 700 nucleotides in length, with a maximal intron size of 3,478 bp (see detailed analysis below). We focused on the genes with the largest introns, with a view to confirming these predictions. For this purpose, 17 introns exceeding 900 bp in length (from 901 to 1,551 bp) were reverse-transcribed and amplified with specific primers and mRNA extracted from cells grown under the three different sets of conditions. Thirteen of these introns were spliced as expected, one was not amplified (cDNA clones revealed a different gene model with no introns), two were found to have been poorly predicted (intron size larger than expected) and the last intron, in YALI0F32043g, was found to be a mosaic of five introns and exons (Additional file 6). Transcripts of this last gene displayed AS due to alternative 3'ss selection (extending exon 4 by 15 bases) and retention of the 60 nucleotides, stop-free intron 5 (Figure 4c; Additional file 5). The observed AS events did not generate in-frame stop codons and did not modify the translation phase. They may result in the generation of different, putatively functional proteins.

**Figure 5:**
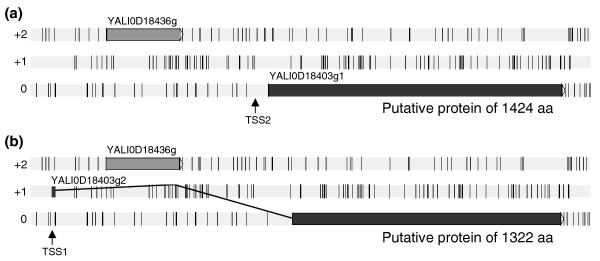
**Schematic diagram of alternative variants of YALI0D18403g**. The two different transcription start sites (TSS1 and TSS2) are indicated by arrows. **(a) **TSS2 is located 179 bases upstream of the methionine initiation codon of YALI0D18403g1 (position 2309045 on chromosome D) downstream of YALI0D18436g and allows the transcription of a single exon. Translation of this mRNA generates a putative protein of 1,322 amino acids. **(b) **TSS1 is located about 3 kb upstream of TSS2 and initiates a transcript with a 3,478-nucleotide intron. Surprisingly, this intron overlaps YALI0D18436g, a CDS of 1,062 bases the translation of which generates a putative 353 amino acid protein of unknown function. Translation of the YALI0D18403g2 mRNAs generates a putative protein of 1,424 amino acids.

Nine additional long introns were detected during the cDNA analysis. The most interesting of these introns was found in YALI0D18403g. Two transcription start sites were found, one located 179 bases upstream of the methionine initiation codon and enabling the transcription of a single exon (Figure 5a), and the other located about 3 kb upstream and giving rise to a transcript with a 3,478-base intron (Figure 5b). Surprisingly, a CDS of 1,062 bases (353 amino acids) of unknown function was predicted within this intron and shown to be highly conserved in the genomes of closely related species (data not shown).

All these results demonstrate the efficient splicing of long introns not necessarily predicted *in silico*.

#### cDNA libraries

**Figure 6:**
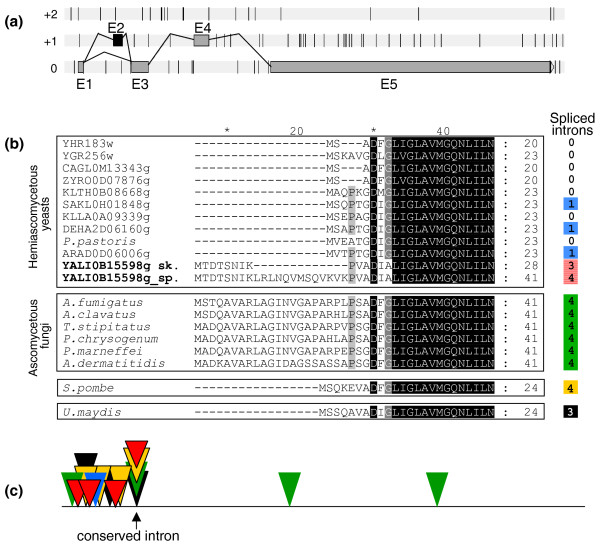
**Alternative splicing in YALI0B15598g and conservation of gene models in *Dikarya *species**. **(a) **Gene models for YALI0B15598g. Exons are represented by gray or black (skipped exon) rectangles and introns by thin black lines. The size of the putative protein is 502 amino acids when intron 1 and intron 2 are efficiently spliced, or 489 amino acids when exon 2 is skipped. **(b) **Amino acid alignment of the amino-terminal domain of fungal and yeast proteins, homologs of YALI0B15598g. The size of this domain is given in amino acids, on the right, for each protein (from 20 to 41). The black rectangle groups together hemiascomycetous yeasts or ascomycetous filamentous fungi. Archiascomycetes are represented by *S. pombe *and basidiomycetes by *Ustilago maydis*. The numbers of spliced introns (column on the right) are colored identically when intron positions are conserved within genes: blue for most hemiascomycetous yeasts, red for *Y. lipolytica*, green for all ascomycetous filamentous fungi, yellow for *S. pombe *and black for *U. maydis. ***(c) **Intron localization. Triangles indicate the position of the introns for the different groups of genes (same colors as in (b)). Only intron 4 of *Y. lipolytica *is conserved in all genes.

The three cDNA libraries were screened for the presence of alternative transcripts and, more specifically, for the presence/absence of the 1,083 introns. Eighty-six introns matched cDNA sequences entirely or partially. For nine of these introns, mRNAs were found in an antisense orientation. Sixty-one of the remaining 75 intron sequences corresponded to the retention of the first (58 cases) or second (3 cases) intron of the gene. Matches for the last 14 intron sequences revealed more complex situations, involving alternative transcription start sites, alternative 5' and 3'ss usage, exon skipping, internal exon and intron retention or combinations of these mechanisms (Additional file 7). For example, in YALI0B15598g, which is highly expressed (24, 9 and 28 cDNA in expo, stat and oleic conditions, respectively), exon 2 was mostly skipped (46 cDNAs versus 2 in which introns 1 and 2 were both efficiently spliced). Exon 2 skipping is facilitated by the presence of suboptimal sequences for intron 1 BP (TGCTCAC) and intron 2 5'ss (GTCAGC). As exon 2 is 39 bp long, both variants encode putative proteins (Figure 6a) homologous to GND1 and GDN2 from *S. cerevisiae*, two 6-phosphogluconate dehydrogenases catalyzing an NADPH-regenerating reaction in the pentose phosphate pathway. These proteins are highly conserved in fungi, with the exception of the amino-terminal domain (Figure 6b). Comparisons of gene models showed the presence of a large number of introns at different sites in the various fungal phyla (Figure 6c). Only intron 4 of YALI0B15598g was found to be conserved in all the basidiomycetes, archiascomycetes and filamentous ascomycetes studied (Figure 6c). Intron 1 of *S. pombe *and *Ustilago maydis *is located at the same position, which differs by few nucleotides from that of *Y. lipolytica *intron 2 or of the single intron retained in some other hemiascomycetous species, such as *Arxula adeninivorans*, *Lachancea kluyveri *and *Debaryomyces hansenii*. Thus, YALI0B15598g may represent an interesting example of intron acquisition or intron slippage.

The different strategies used to detect alternative transcripts in *Y. lipolytica *revealed that such variants were generated from at least 88 genes (Additional files 7 and 8). All known modes of AS were observed: alternative 5'ss (3 genes) and 3'ss usage (6 genes), exon skipping (4 genes) and intron retention (76 genes). Alternative transcription start sites within or downstream of introns were detected in seven genes. Alternative transcripts were observed for 9.2% of the intron-containing genes, but for only 1.8% of these genes if intron retention was excluded. Most of the variants observed resulted from intron retention and, if only multi-intronic genes were considered, the intron retained was mostly the first intron (15 of 21 genes). In almost all cases, intron-containing transcripts revealed by our experimental approaches, carried a PTC. This type of mRNA is generally detected by the NMD pathway, a quality control mechanism that recognizes and degrades PTC-containing transcripts, preventing their translation.

### The NMD machinery exists in *Y. lipolytica*, but some effectors are lacking

The presence and efficiency of NMD was investigated in *Y. lipolytica*. Homologs of *UPF1 *(*YlUPF1*, YALI0D23881g) and *UPF2 *(*YlUPF2*, YALI0E24629g) were detected in the *Y. lipolytica *genome by searches for similarity to known genes. *UPF3*, which is less well conserved in eukaryotes than *UPF1 *or *UPF2*, was not detected in the chromosomes or in any non-assembled reads, suggesting that this NMD effector is lacking or highly divergent in *Y. lipolytica*. We also looked for *SMG1*, *SMG5*, *SMG6 *and *SMG7 *(*EBS1 *in *S. cerevisiae*), but failed to detect any homologs in *Y. lipolytica*.

The YALI0D23881g and YALI0E24629g genes, encoding *YlUPF1 *and *YlUPF2*, were entirely deleted from the laboratory strain PO1d. None of the single-deletion mutants for these genes displayed a growth defect under the conditions tested, and no defect was observed for the double-mutant (Figure S5 in Additional file 2). This result is consistent with the absence of a growth defect in *S. cerevisiae *strains lacking the *UPF1 *or *UPF2 *gene [68,69], suggesting that NMD is not an essential biological mechanism in yeasts.

**Figure 7:**
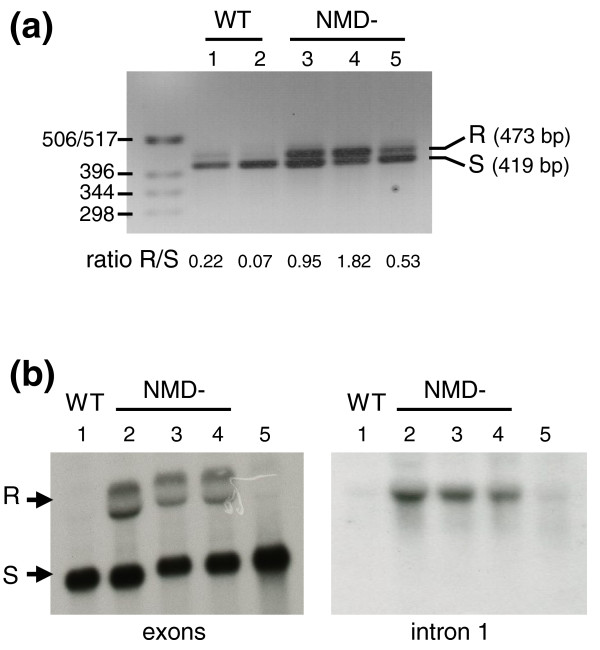
**Gene expression in the NMD^- ^context**. **(a) **Variations in the level of expression of YALI0C23496g splicing variants as a function of NMD context. RT-PCR products from spliced (S) and unspliced transcripts (intron 1 retained, R) from wild-type strains (WT) and NMD mutants (NMD-). Wild-type strains are E150 (lane 1) and PO1d (lane 2). NMD^- ^strains are two independent knockouts of *UPF1 *(lane 3, *upf1*::*LEU2 *clone 7; lane 4, *upf1*::*LEU2 *clone C) and one *UPF2 *knockout (lane 5: *upf2*::*LEU2 *clone 7). The intensity of the unspliced transcripts is much stronger in the mutant strains. **(b) **Expression of the different transcripts of the *Y. lipolytica YRA1 *gene. Northern blot of total RNA of wild-type (WT) strain PO1d (lane 1) and NMD^- ^mutant strains *upf1*::*LEU2 *clone 7 (lane 2), *upf2*::*LEU2 *clone 7 (lane 3), *upf1*::*URA3 upf2*::*LEU2 *(lane 4), *xrn1*::*LEU2 *(lane 5). The exon probe binding to exons 1 and 3 reveals the spliced transcript (S) in all strains and an additional splicing variant in NMD^- ^mutants only. This variant corresponds to the retention of intron 1 (R). Hybridization with intron 1 confirmed that this intron is retained only in NMD^- ^mutants, whereas it is efficiently spliced out in PO1d and xrn1^- ^mutants.

The efficiency of NMD in *Y. lipolytica *was assessed by comparing the levels of PTC-containing transcripts in wild-type and mutant strains. RT-PCR was performed on four populations of mRNAs (YALI0B011154g, YALI0C23496g, YALI0D05041g and YALI0F16752g) displaying intron retention and resulting in the generation of a PTC, and four populations of efficiently spliced mRNAs (YALI0B15598g, YALI0E20031g, YALI0F03179g and YALI0F09669g). In the efficiently spliced mRNAs, we found no difference in the ratio of efficiently spliced to unspliced transcripts between the wild-type and the mutant strains (Figure S6 in Additional file 2). Among the second set of genes, YALI0D05041g and YALI0F16752g showed a very low level of intron retention, which did not increase in NMD mutants (Figure S6 in Additional file 2). This observation suggests that both genes are probably not subjected to the NMD pathway. In contrast, for YALI0C23496g and YALI0B11154g the ratios of spliced and unspliced transcripts clearly differed between wild-type and mutant strains. The intensity of the RT-PCR product for the unspliced transcript was clearly higher in NMD mutants (the unspliced/spliced (R/S) ratio increased from 0.09 to 0.35 for YALI0B11154g and from 0.07 to 1.82 for YALI0C23496g; Figure 7a). Thus, despite the lack of a conserved *UPF3 *homolog, NMD is functional in *Y. lipolytica *and unspliced transcripts of YALI0C23496g and YALI0B11154g are targeted by this degradation pathway.

We also focused on the homolog of YDR381W (*YRA1*), which is known to encode an mRNA not targeted by the NMD pathway in *S. cerevisiae *[41,42]. Homologs of the *YRA1 *gene are conserved in all hemiascomycetous yeasts and present a long intron, the BP motif of which diverges from the canonical sequence in almost all species [41]. The *Y. lipolytica *gene model for the *YRA1 *homolog (YALI0A20867g) follows this rule: the first intron is 850 bp long and its BP sequence, located three bases upstream of the 3'ss motif, is TGCTGAC. RT-PCR validated the splicing of the intron in wild-type and mutant strains but identified no transcripts in which intron 1 was retained. However, as the difference in the lengths of the spliced and unspliced forms may bias the PCR in favor of the spliced variant, northern blots were performed with probes binding to exons or the first intron (Figure 7b). Given that *YRA1 *mRNA degradation requires Xrn1p in *S. cerevisiae *[42], we also included a *YlXRN1 *mutant in our analysis. In hybridization studies, we observed a higher intensity of the bands corresponding to intron-retained transcripts in NMD^- ^mutants only. No such increase in intensity was observed for the *YlXRN1 *mutant. This observation suggests that, as in *S. cerevisiae*, the *YlYRA1 *transcript is not efficiently spliced in *Y. lipolytica*. We also found that, by contrast to what has been reported for *S. cerevisiae*, unspliced transcripts were targeted by the NMD pathway, and their degradation seemed to be independent of the YlXrn1 protein. These results suggest that the *S. cerevisiae YRA1 *autoregulation mechanism based on the nuclear export and cytoplasmic Edc3p-mediated decay of the unspliced transcript [42], is probably not conserved in *Y. lipolytica.*

## Discussion

Hemiascomycetous yeasts are considered to have intron-poor genomes. We show here that despite this intron paucity, *Y. lipolytica *has four times as many introns as *S. cerevisiae *and is the hemiascomycetous genome with the largest number of intron-containing genes sequenced to date. The combination of approaches used made it possible to correct many predicted gene models, to identify new genes, such as *SOA *genes [51], to confirm the splicing of many introns, including both large introns and introns from weakly expressed genes, and to detect introns in 5' UTRs. From a structural point of view, the genome annotation of *Y. lipolytica *is now largely validated by experimental data and provides a reliable genome model complementary to that of *S. cerevisiae*.

We show here that *Y. lipolytica *produces alternative transcripts through several different mechanisms: intron retention, exon skipping, 3' and 5' alternative splice site usage and the use of alternative promoters. The frequency of AS in *Y. lipolytica *is not very high, particularly if intron retention is excluded from the analysis (1.8% of intron-containing genes), but remains higher than that reported for *S. cerevisiae *or other hemiascomycetous yeasts, in which few naturally occurring cases [16,26,27,29,31,38,39] or experimentally induced examples [70,71] have been described. Additional cases have been detected in yeasts, thanks to the recent development of genome-wide technologies providing information about transcript polymorphism, such as tiling or RNA-seq approaches, but these cases mostly involve intron retention [32-34]. We report here a few interesting examples of exon skipping, alternative 3'ss usage or presence of an intronic gene, the expression of which depends on an alternative promoter. The situation is quite different in basidiomycetous yeasts, such as *Cryptococcus neoformans*, which has an intron-rich genome (mean of 5.3 introns per gene) and a high frequency of AS, with high levels of intron retention [72] but 4.2% of the transcripts nonetheless resulting from exon skipping and alternative 3'ss or 5'ss usage [73].

In *Y. lipolytica*, intron retention is the main model by which mRNA variants are generated, consistent with previous findings for ascomycetous fungi [74,75]. However, the particular involvement of the first intron in intron retention has not been reported before, probably because AS was investigated mostly in hemiascomycetous yeasts with very few multi-intronic genes [14]. It would be interesting to perform a similar analysis in other phyla of ascomycetous fungi or in basidiomycetes. If this phenomenon reflects an ancestral trait, then the bias should be more marked in filamentous fungi known to possess intron-rich genomes.

One of the key questions emerging from our study relates to whether intron retention in *Y. lipolytica *plays a physiological role, as observed for *YRA1 *or meiotic genes in *S. cerevisiae*, or reflects an underlying background of splicing failure. We addressed this question by investigating whether the retained introns were different from other introns, including, specifically, whether their 5'ss, 3'ss or BP were degenerate or whether the introns were particularly long, potentially accounting for the low splicing efficiency (Additional file 8). However, no bias was detected in primary structure, except that the first intron of *YRA1 *had a degenerate BP, as it does in *S. cerevisiae *[41]. More recently, *YRA1 *splicing inhibition has been reported to be regulated by *YRA1 *exon 1, in a size-dependent but sequence-independent manner [42]. We thus investigated the size of exon 1 (coding exon plus 5' UTR) in inefficiently spliced transcripts but, again, no bias was detected. Another possibility, requiring further investigation for *Y. lipolytica *introns, stems from the reported correlation between splicing efficiency and the spatial distance between 5'ss and BP [76,77]. It has been suggested that a zipper stem in the secondary structure of three large introns of *S. cerevisiae *shortens the S1 distance and facilitates spliceosome assembly [77].

However, as the first intron is more often retained than downstream introns, it is tempting to speculate that, in most cases, intron retention probably results from a defect in the kinetics of spliceosome recruitment by the polymerase or in spliceosome assembly. Indeed, in *S. cerevisiae *[78], as in other eukaryotes, splicing is mostly cotranscriptional [79]. It has also been shown that the efficiency of splicing factor recruitment during transcription may influence splicing efficiency [80] and that the carboxy-terminal domain (CTD) of the large subunit of polymerase II is involved in this mechanism. We can thus speculate that retention of the first intron of transcripts may result from a defect in the recruitment of the spliceosome by polymerase II during transcription. We are currently investigating this hypothesis in *Y. lipolytica *and determining whether there is a correlation between intron retention and the binding kinetics of introns, splicing factors and CTD.

Almost all the observed unspliced transcripts included a premature termination codon. During the first round of translation (before degradation by NMD), the ribosome is thus likely to be rapidly stopped by the PTC because the introns concerned were mostly located at the 5' end of the CDS, close to the start codon. A statistical analysis of the structural characteristics of *Y. lipolytica *introns (intron size, frame of integration within the coding sequence, PTC) revealed that up to 93% of introns generated a PTC, whereas only about 30% of introns in *Paramecium *generate PTCs [50]. This high percentage is due to both intron size and, in half the cases, the sequence of intron boundaries. The presence of stop codons in 5'ss motifs is unusual for yeast introns, as the most frequent motif in the hemiascomycete genomes sequenced to date is GTATGT [14,61], but the 5'ss motif in *Y. lipolytica *is GTGAGT. This observation highlights a specific evolution of intron features in *Y. lipolytica*, as proposed for various intron-poor lineages with strong 5'ss [60]. Introns of size 3n were also found to be underrepresented, and stop-free 3n introns were particularly strongly underrepresented, as previously reported for other eukaryotes [50]. If retained, 3n stop-free introns do not change the translation frame and are thus considered as coding sequences that may affect the structure and activity of the resulting protein, with possible deleterious consequences for the cell. In *Y. lipolytica*, the small number of 3n introns was particularly pronounced for short introns, probably reflecting an ancestral situation in which introns were numerous and short [20]. Intron size has increased during the course of evolution in yeasts, including *Y. lipolytica *in particular, to a much greater extent than in filamentous ascomycetes. This size increase has increased the likelihood of introns containing a PTC, potentially limiting the need for specific constraints on intron size (3n, 3n + 1, 3n + 2).

In eukaryotes, mRNAs with PTCs are subject to NMD, a quality-control mechanism directing PTC-containing transcripts for degradation to prevent their translation (for reviews, see [43-45]). As most *Y. lipolytica *transcripts containing retained introns also contain PTCs, this would suggest that such transcripts are mostly targeted by NMD. This may account for their lack of detection in assays with wild-type strains, in which they were probably degraded by the NMD pathway too rapidly for detection. We therefore investigated whether NMD was active in *Y. lipolytica*. Genes for only two of the core NMD factors were detected in the sequenced strain, *YlUPF1 *and *YlUPF2*. This situation is exceptional among eukaryotes, as all other organisms in which NMD has been studied possess at least three major effectors (*UPF1*/*SMG2*, *UPF2*/*SMG3 *and *UPF3*/*SMG4*). Deleting *YlUPF1 *or *YlUPF2 *resulted in a significant increase in the proportion of unspliced transcripts for some, but not all intron-containing genes. This result confirms the existence of a functional NMD pathway in *Y. lipolytica*. However, the absence of significant growth defects in *YlUPF1 *and *YlUPF2 *mutants suggests that NMD is not an essential mechanism, as in *S. cerevisiae *and *Caenorhabditis elegans*, whereas it has been shown to be essential in plants and metazoans (for a review, see [43]). We now aim to determine, at the whole-genome scale, which genes are targets of NMD and how this pathway is regulated in this yeast.

## Conclusions

We present here an extensive survey of the transcriptome of a yeast chosen for this study on the basis of its phylogenetic position, far removed from all other hemiascomycetous yeasts sequenced to date. This in-depth analysis of the transcriptome made it possible to improve the structural annotation of the *Y. lipolytica *genome and identified complex cases of alternative transcripts. With a genome slightly more complex than that of *S. cerevisiae *in terms of gene structure, together with its genetic and biochemical tractability, *Y. lipolytica *may be a valuable organism for studies of the regulation of AS and its impact on the evolution of gene structure. Although considered an intron-poor species, *Y. lipolytica *nonetheless displays significant biases in its intron structure, generating PTCs in cases of intron retention. However, further comparative studies at a larger phylogenetic scale are clearly required to determine whether the modeling of intron-containing genes corresponds to an ancestral characteristic or to an evolutionary phenomenon acquired in this particular lineage.

## Materials and methods

### Strains and media

*Y. lipolytica *strains E150 (CLIB122, MATB his-1 leu2-270 ura3-302 xpr2-322) and PO1d (CLIB139, MatA leu2-270 ura3-302 xpr2-322) were routinely grown at 28°C on YPD (yeast extract, peptone and glucose, 10 g/l each) or YNB (1.7 g/l Yeast Nitrogen Base (Difco, Detroit, MI, USA), 10 g/l glucose) supplemented for auxotrophy if necessary. Oleic acid medium was prepared as follows: 1.7 g/l Yeast Nitrogen Base (Difco), 50 g/l NH_4_Cl, 50 mM PO_4_NaK pH 6.8, a 100 ml/l emulsion of oleic acid (oleic acid 20% (v/v), 0.625% (v/v) Tween 40), 0.8 g/l yeast extract, 10 g/l glucose. Growth phenotypes were investigated for mutant strains of PO1d, on YPD and YNB medium, at 28°C and 18°C.

#### RNA extraction and RT-PCR

The RNeasy Midi Kit (Qiagen, Courtaboeuf, France) was used to extract total RNA from cells grown in three different conditions: exponential growth phase in YPD (called 'expo'), stationary phase in YPD ('stat') and exponential growth phase in oleic acid medium ('oleic'). DNA contamination was eliminated with the Turbo DNA-free kit (Applied Biosystems/Ambion, Austin, Texas, USA). RT-PCR was performed with Ready-To-Go™ RT-PCR Beads (GE Healthcare Life Sciences, Orsay, France) and PCR control with PuReTaq Ready-To-Go™ PCR Beads (GE Healthcare Life Sciences). Primers for RT-PCR were designed so as to obtain a 200-bp amplicon after splicing. The resulting amplicons were subsequently inserted into a Bluescript plasmid and sequenced to identify the different splicing variants. The relative intensities of RT-PCR products were estimated from ethidium bromide-stained gels, with the ImageJ software developed at the National Institutes of Health [81].

#### Northern blotting

About 20 μg of RNA was separated by electrophoresis in a 1.2% agarose gel in 1× FA buffer (20 mM morpholinepropanesulfonic acid, 5 mM sodium acetate, 1 mM EDTA, pH 7) supplemented with 1.8% formaldehyde. After electrophoresis, the RNAs were transferred onto GeneScreen nylon membranes (Perkin-Elmer Life Sciences, Courtaboeuf, France), as previously described [82]. DNA probes were amplified by PCR from the genomic DNA of strain E150. PCR products were purified by electrophoresis in a 1% low-melting point agarose gel. DNA probes were labeled with [α-^32^P]dCTP, with the Amersham Megaprime™ DNA labeling kit (GE Healthcare Life Sciences, Orsay, France), and hybridizations were performed in Denhardt's solution-containing buffer at 65°C [83]. Final washes were performed at 65°C, in 0.2 × SSC (1 × SSC is 0.15 M NaCl plus 0.015 M sodium citrate)-0.1% sodium dodecyl sulfate.

#### cDNA library construction and sequencing

Total RNA was extracted from cells grown in three different sets of culture conditions (expo, stat and oleic; see above). We isolated mRNA from the total RNA preparation with the Oligotex mRNA kit (Qiagen) and the three libraries were constructed with the CloneMiner™ cDNA Library Construction Kit (Invitrogen, Cergy Pontoise, France), based on Gateway^® ^technology. The resulting libraries were highly enriched in full-length, oriented clones. We sequenced 28,434 clones (9,409, 9,620 and 9,405 clones for the expo, stat and oleic libraries, respectively) by the Sanger method, first from the 5' end of the cloning cDNAs and then from the 3' end for 1,414 chosen clones. For 1,004 of these 1,414 selected clones, 5' and 3' sequences have been assembled, whereas for the remaining 410 clones, the 5' and 3' sequences were deposited individually in the EMBL database. The accession numbers for the resulting 28,844 sequences are [EMBL:FP671140-EMBL:FP680548], [EMBL:FP680607-EMBL:FP690338] and [EMBL:FP690350-EMBL:FP700052] for the expo, stat and oleic libraries, respectively.

#### Gene deletion

The complete deletion of *Y. lipolytica *genes (YlUPF1, YALI0D23881g; YlUPF2, YALI0E24629g; YlXRN1, YALI0C23144g) was performed as previously described [84]. Primers for the PCR amplification of promoter (P) and terminator (T) regions are listed in Additional file 9. The ML and/or MU cassettes [84] were introduced into the PT cassette. PO1d cells were transformed by the lithium acetate method [85], with about 400 ng of purified DNA from the disruption cassettes. Transformants were selected on YNB medium supplemented with NH_4_Cl (5 g/l), sodium potassium phosphate buffer, pH 6.8 (50 mM), agar (2%) and uracyl (100 mg/ml) or leucine (100 mg/ml). Gene deletion was checked by PCR, with primers external to the disruption cassette, upstream from P and downstream from T.

Auxotrophic mutants were complemented with the *URA3 *or *LEU2 *cassettes, for comparison of their growth rates with that of the wild-type strain, W29.

#### Genome sequence and sequence analysis

At the beginning of this study, the genome annotation of *Y. lipolytica *strain E150 included 6,703 CDSs (genes and pseudogenes [1]) and 742 introns (First annotation version 3 July 2004). The genomic sequence and the different versions of the genome annotation of *Y. lipolytica *strain E150, including the version updated with our data, are available from the Génolevures database [52].

The sequences of the cDNA clones were compared with sequences in a nucleotide sequence database of *Y. lipolytica *CDS using BLAST [86]. Only the first hit was considered if the expected value was lower than 1.e-100 or between 1.e-50 and 1.e-100 with an identity score exceeding 95%.

DNA logos were created with WEBLOGO version 2.8.1 [87,88].

## Abbreviations

3'ss: 3' splice site; 5'ss: 5' splice site; AS: alternative splicing; bp: base pair; BP: branch point; CDS: coding sequence; CTD: carboxy-terminal domain; NMD: nonsense-mediated mRNA decay; PTC: premature termination codon; UTR: untranslated region.

## Authors' contributions

CN conceived and designed the experiments; MM, IBL, CO and CN performed the experiments. CC, CDS and PW performed the cDNA sequencing; CN and MM analyzed the data; CN and CG wrote the paper. All authors read and approved the final manuscript.

## Supplementary Material

Additional file 1 **Supplementary Table S1**. Click here for file

Additional file 2 **Supplementary figures**. Click here for file

Additional file 3 **Supplementary Table S2**. Click here for file

Additional file 4 **Supplementary Table S3**. Click here for file

Additional file 5 **Supplementary Table S4**. Click here for file

Additional file 6 **Supplementary Table S5**. Click here for file

Additional file 7 **Supplementary Table S6**. Click here for file

Additional file 8 **Supplementary Table S7**. Click here for file

Additional file 9 **Supplementary Table S8**. Click here for file
