# Supplementary material for: Detection and analysis of alternative splicing in Yarrowia lipolytica reveal structural constraints facilitating nonsense-mediated decay of intron-retaining transcripts
Source: Genome Biol. 2010 Jun 23;11(6):R65. doi: 10.1186/gb-2010-11-6-r65 (PMC2911113; doi:10.1186/gb-2010-11-6-r65)
Supplement: Additional file 2 — Supplementary figures. [file gb-2010-11-6-r65-S2.PDF]

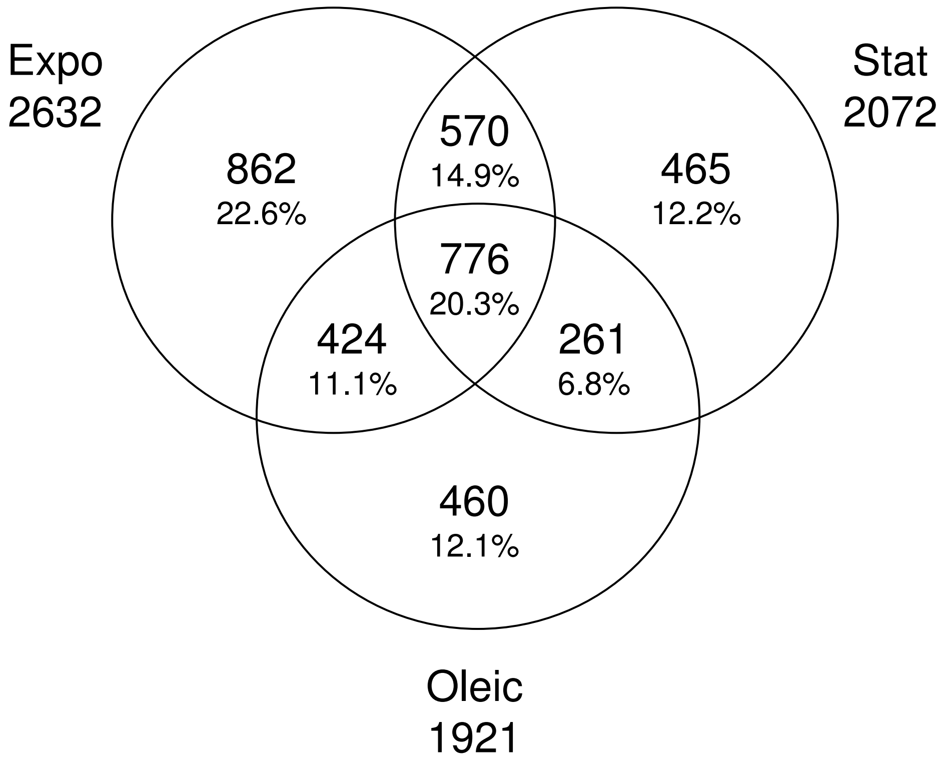

Supplemental figure 1: Occurrence of *Y. lipolytica* genes and repartition in the three cDNA libraries. Percentages indicate the proportion of genes among the 3818 genes identified in all cDNA clones. Expo: exponential phase in YPD medium, Stat: stationary phase in YPD medium, Oleic: exponential phase on oleic acid medium.

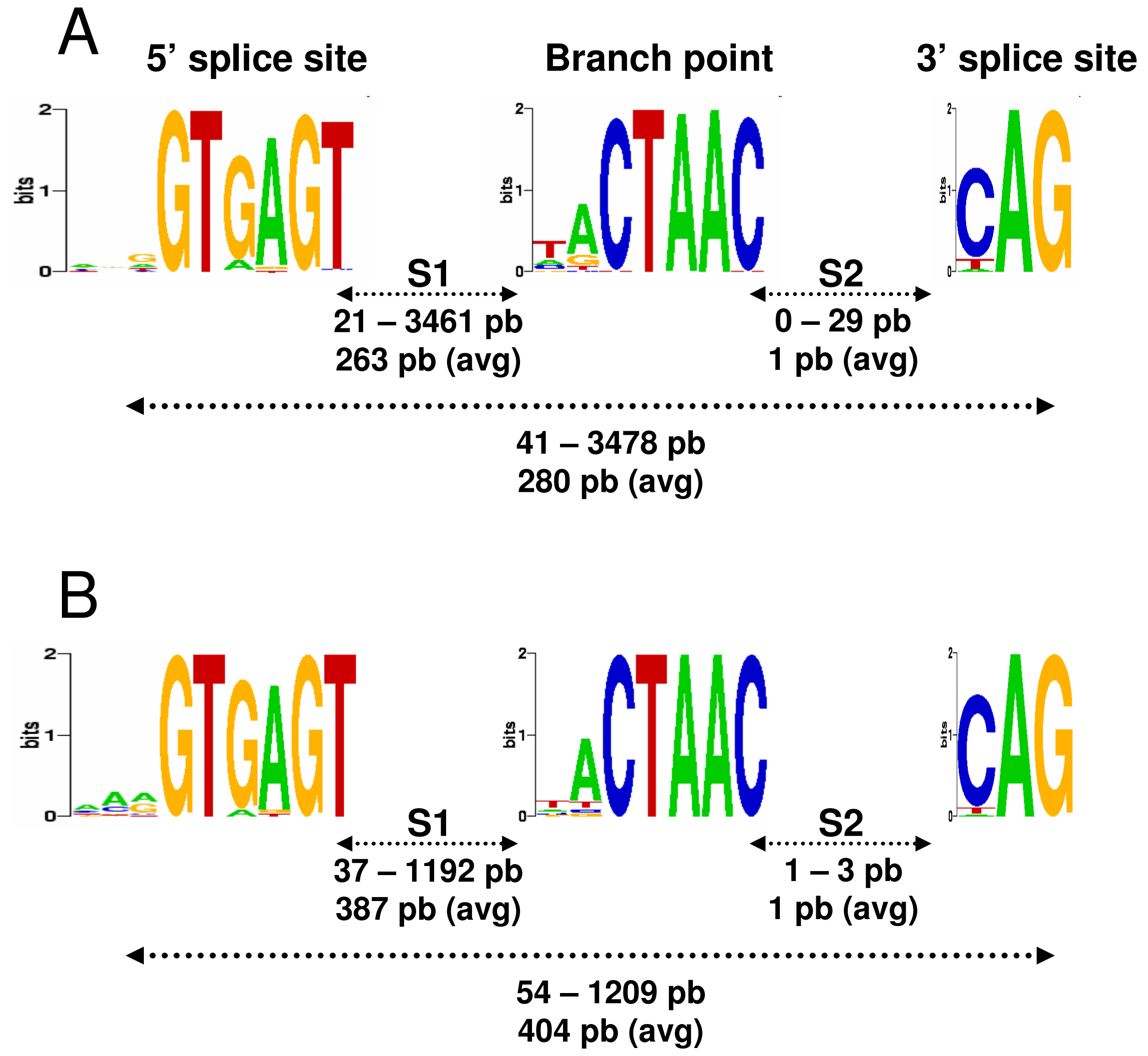

Supplemental figure 2: Intron pattern of *Y. lipolytica* strain E150. Consensus sequences for 5' splice site, branch point, 3' splice site and for the 3 nucleotides upstream introns are displayed with a logo representation (Schneider and Stephens, 1990). S1 and S2 represent the distances between 5'ss consensus motif and branch point (BP) motif, and between BP and 3'ss motifs, respectively. Minimal and maximal lengths, as well as mean size of S1, S2 and intron are reported below dotted arrows.

A. Pattern of introns located within the coding exons determined from 1070 predicted introns.

B. Pattern of introns located within the 5'UTR determined from 36 introns.

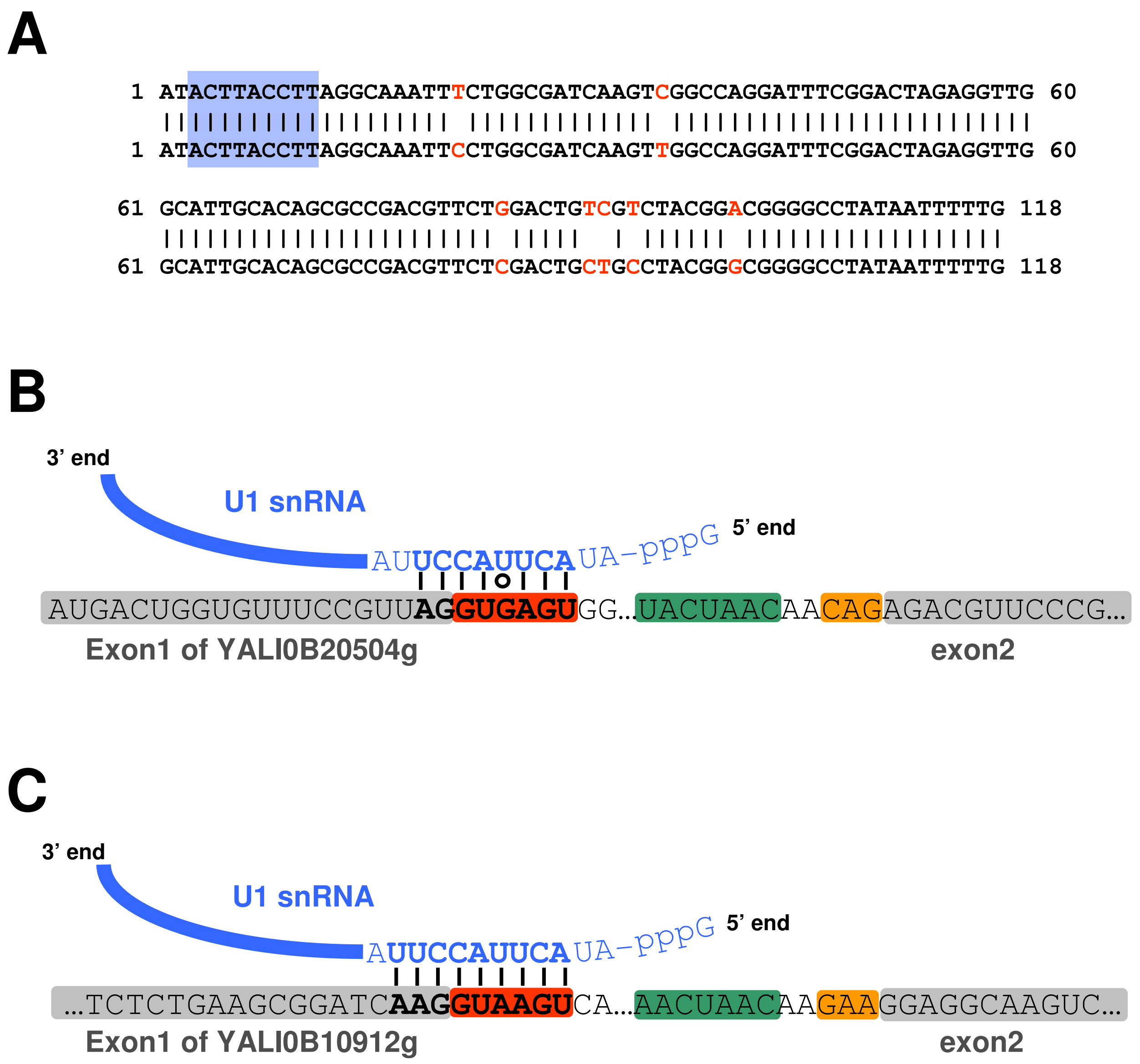

Supplemental figure 3: snRNA U1 genes and base-pairing with 5’-splice site motifs.

A. Alignment between YALI0B14567r and YALI0B20936r, both encoding snRNA U1. Nucleotide identity between both genes is 93.75%. Divergent nucleotides are in red. The blue rectangle highlights nucleotides corresponding to the base-pairing region.

B. Base-pairing between snRNA U1 (in blue) and the 5’-splice site of the first intron of YALI0B20504g. In this case the 5’-splice site (in a red box) is GUGAGU, the major 5’ss motif (87% of the introns) used by *Y. lipolytica* introns.BP and 3’-splice site motifs are in green and yellow boxes, respectively.

C. Same as **B** with the first intron of YALI0B20504g. Here the 5’-splice site is GUAAGU, the second major 5’ss motif (6.2% of the introns) used by *Y. lipolytica* introns.

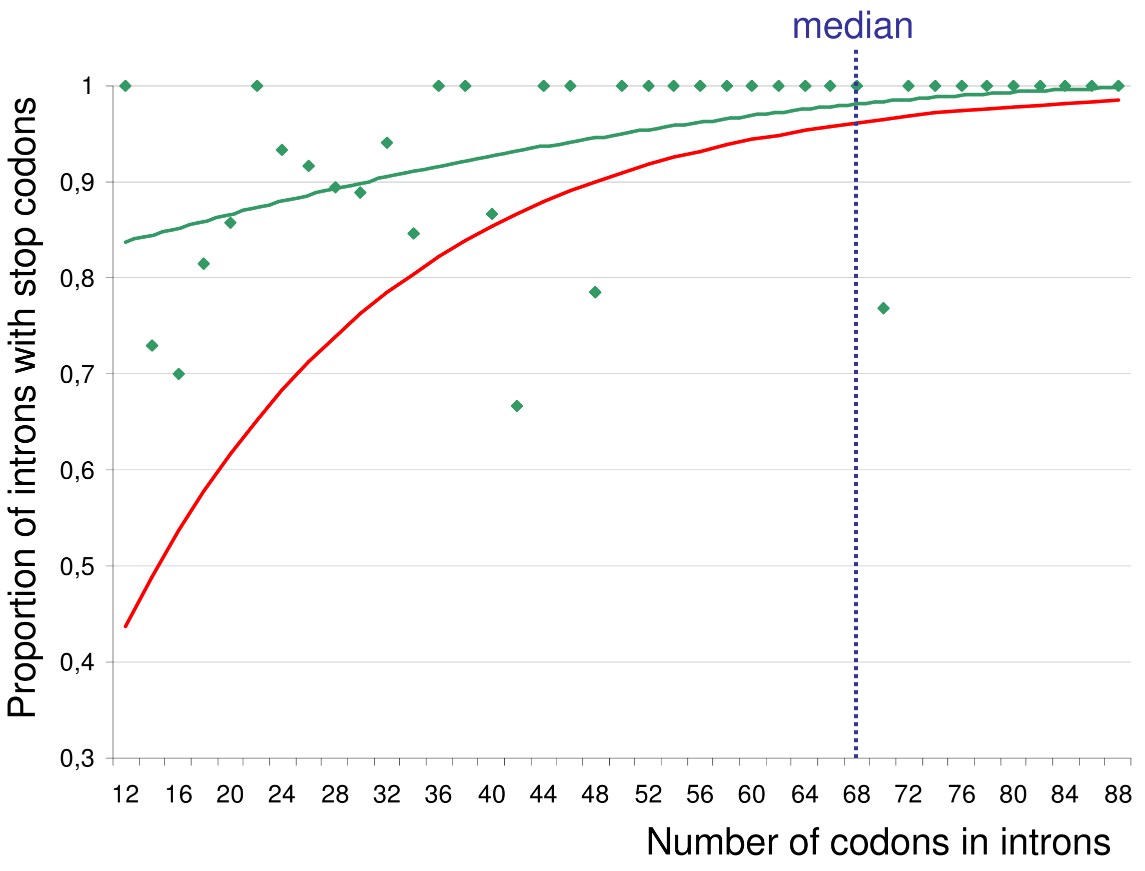

Supplemental figure 4: Statistical significance of the high proportion of PTC-containing introns. Comparison between expected (red curve) and observed (green dots and trend curve) proportions of introns that contain PTC. The median for intron length (204 nt) is represented by a dotted blue line. The expected curve was calculated for random chains of codons of the length shown in abscissa. Assuming a GC content of 50% and random choice of codons, the probability (*p*) of carrying an in-frame stop codon is $p = 1\text{-}(61/64)^{\mathbf{n}}$ where n = number of codons. For introns larger than 186 nt (with more than 62 codons), *p* exceeds 0.95. The trend curve associated with the observed data is the following polynomial function: $y = -9e\text{-}^{05}x^2 + 0.0078x + 0.8295$. This graph highlights the higher than expected proportion of PTC-containing introns in the 12-68 codon size range corresponding to 50% of the introns. The correlation between both curves is -0,425.

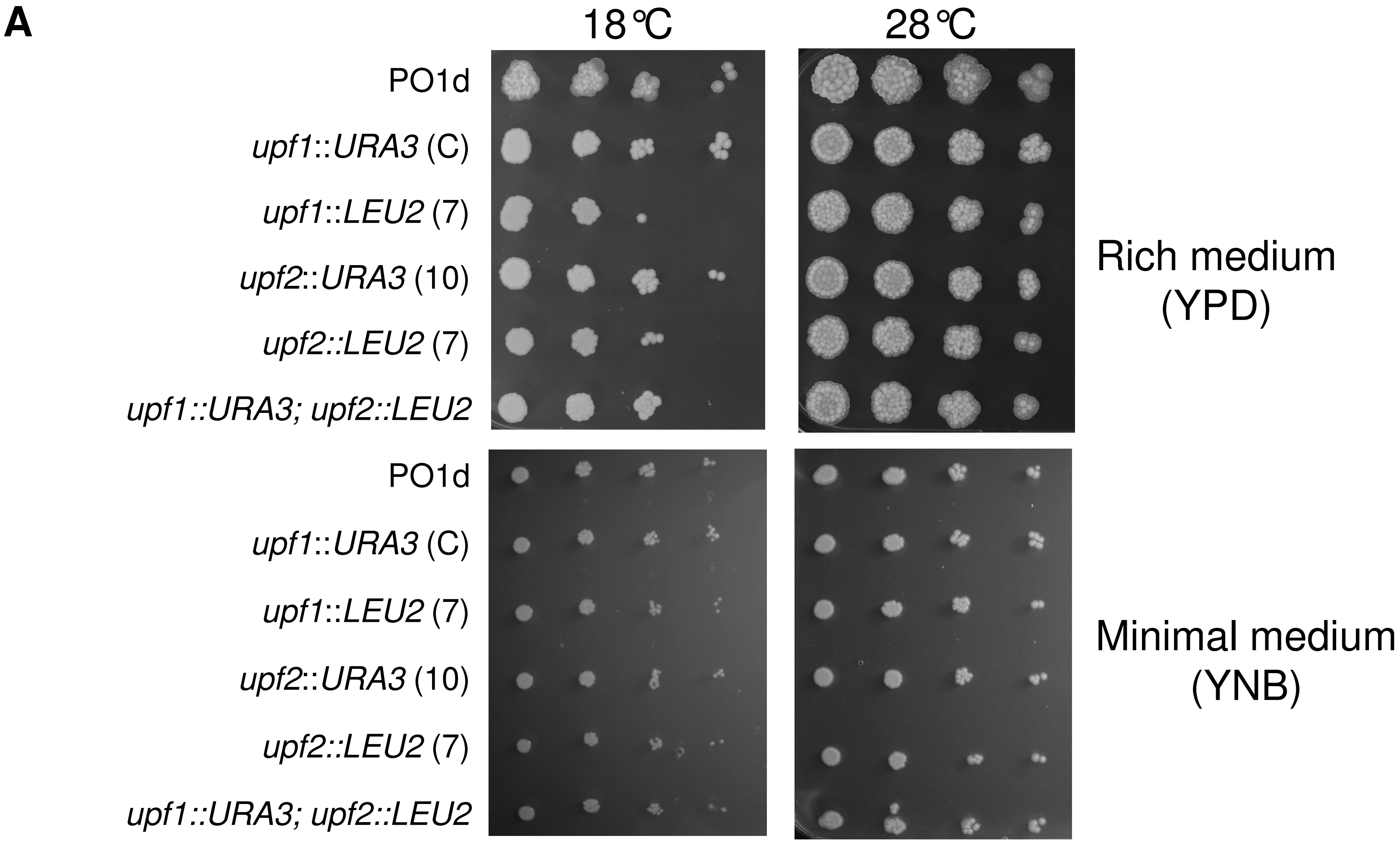
A
18℃
28℃
PO1d
upf1::URA3 (C)
upf1::LEU2 (7)
upf2::URA3 (10)
upf2::LEU2 (7)
upf1::URA3; upf2::LEU2
Rich medium (YPD)
PO1d
upf1::URA3 (C)
upf1::LEU2 (7)
upf2::URA3 (10)
upf2::LEU2 (7)
upf1::URA3; upf2::LEU2
Minimal medium (YNB)

B

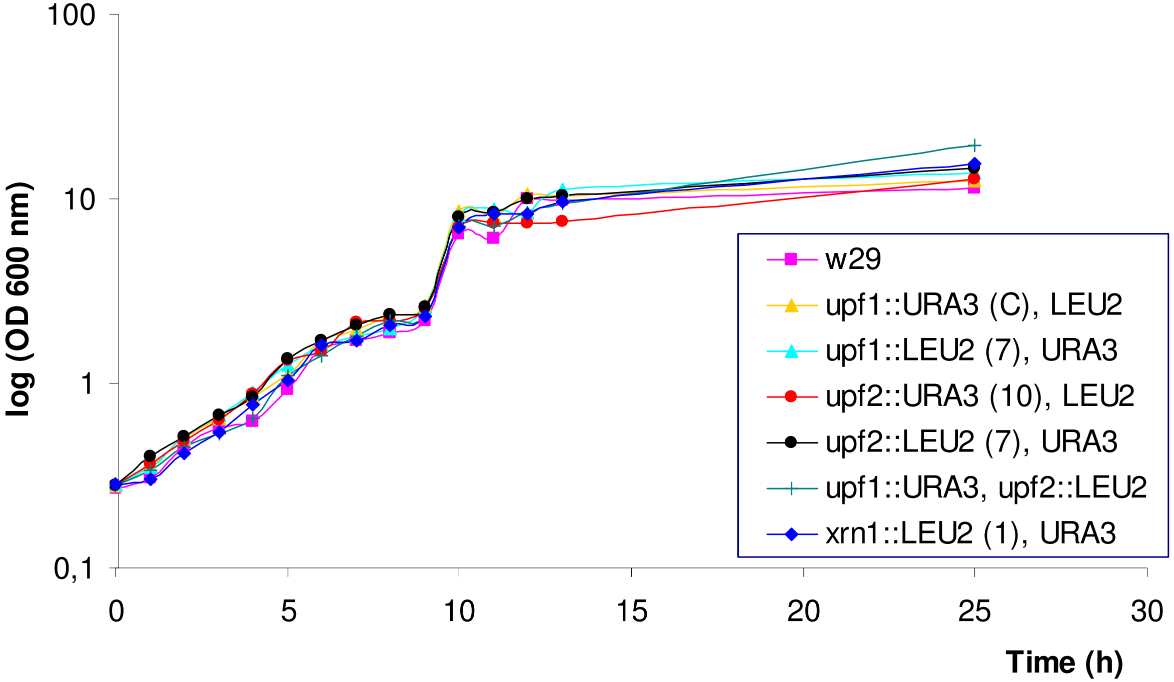

Supplemental figure 5: Growth phenotype of *Y. lipolytica* strains deleted for NMD factors. No growth defect has been detected in the NMD (UPF1, UPF2) and mRNA degradation (XRN1) mutants.

A. Growth test on agar plate with rich (YPD) or minimum (YNB) medium at 18°C and 28°C.

B. Growth curve on YPD rich medium at 27°C. All auxotrophic mutants have been complemented in order to be compared to the prototrophic wild type strain W29.

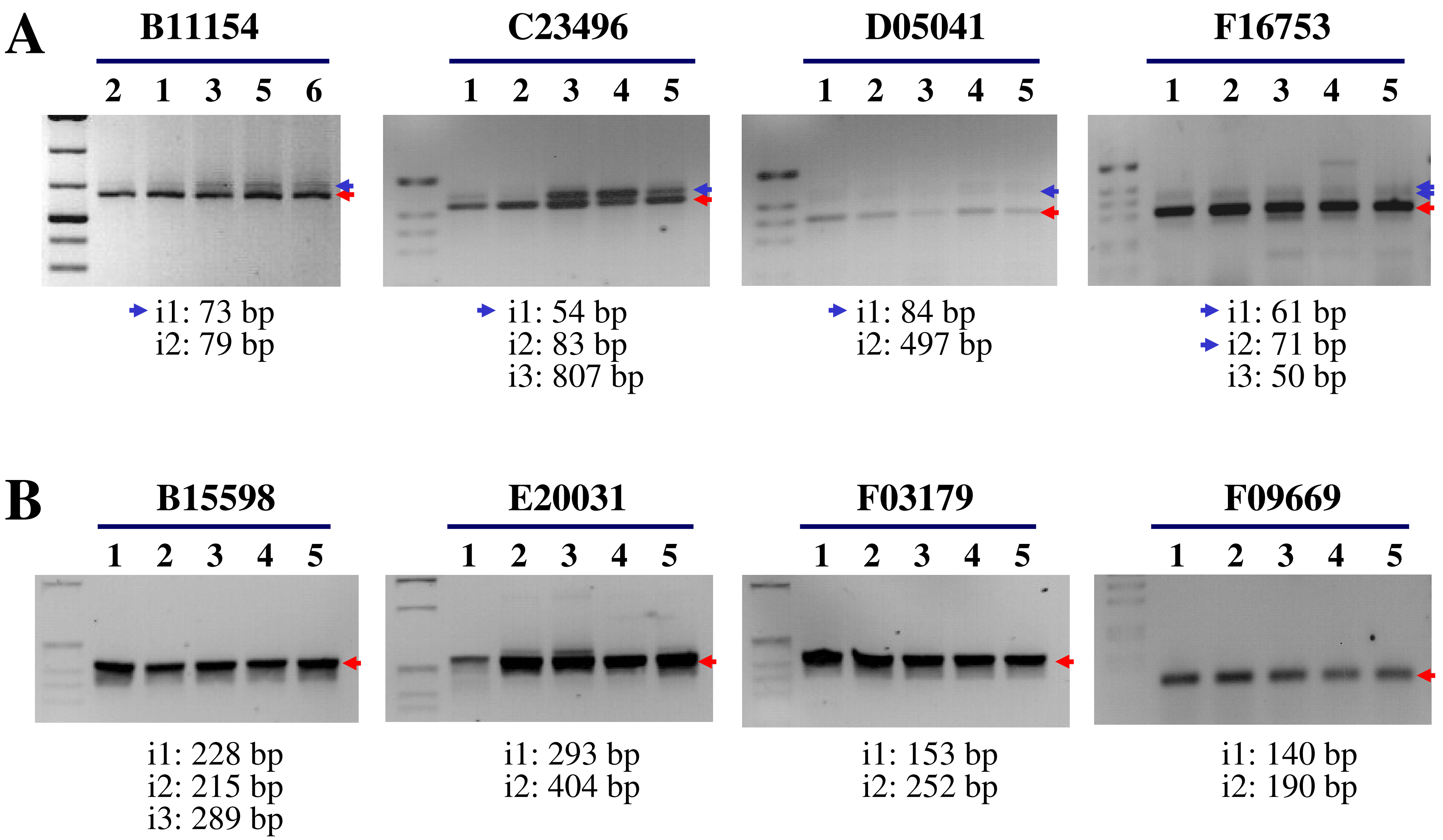

Supplemental figure 6: Spliced transcripts revealed by RT-PCR in wild type and NMD mutant strains.

A. Retention of intron 1 of four different genes was first evidenced by RT-PCR in the wild type strain E150. Abbreviation of the gene names (B07205 for YALI0B07205g) is given above each RT-PCR. Red and blue arrows indicate spliced and unspliced mRNAs, respectively.

B. Introns of these four genes were first found efficiently spliced in the wild type strain E150.

1: E150, **2**: PO1d, **3**: *upf1*::*LEU2* clone 7, **4**: *upf1::LEU2* clone C, **5**: *upf2::LEU2* clone 7, **6**: *upf1::URA3 upf2::LEU2*. Intron size is given below each RT-PCR (i1: intron 1, i2: intron2, i3: intron 3).
